# Platinum resistance in breast and ovarian cancer cell lines

**DOI:** 10.1186/1756-9966-30-91

**Published:** 2011-10-04

**Authors:** Niels Eckstein

**Affiliations:** 1 Federal Institute for Drugs and Medical Devices, Kurt-Georg-Kiesinger-Allee 3, 53175 Bonn, Germany

## Abstract

Breast and ovarian cancers are among the 10 leading cancer types in females with mortalities of 15% and 6%, respectively. Despite tremendous efforts to conquer malignant diseases, the *war on cancer *declared by Richard Nixon four decades ago seems to be lost. Approximately 21,800 women in the US will be diagnosed with ovarian cancer in 2011. Therefore, its incidence is relatively low compared to breast cancer with 207.090 prognosed cases in 2011. However, overall survival unmasks ovarian cancer as the most deadly gynecological neoplasia. Platinum-based chemotherapy is emerging as an upcoming treatment modality especially in triple negative breast cancer. However, in ovarian cancer Platinum-complexes for a long time are established as first line treatment. Emergence of a resistant phenotype is a major hurdle in curative cancer therapy approaches and many scientists around the world are focussing on this issue. This review covers new findings in this field during the past decade.

## Introduction

Among solid gynaecological tumors, breast cancer is the most often diagnosed tumour while ovarian cancer is the most deadly gynaecological neoplasia. Cisplatin plays a completely different but important role in the treatment of both female cancer types. In ovarian cancer treatment, Platinum-based chemotherapy plays a pivotal role as first line chemotherapy option and is usually combined with taxanes [1]. In breast cancer treatment, cisplatin yet only is regarded a cytostatic reserve. According to current guidelines, treatment of breast cancer normally is performed as chemotherapy triplets. The most commonly used cytostatics in the clinical management of the disease are Anthracyclines, Cyclophosphamide, Fluorouracil, and Taxanes, respectively. Prominent examples of chemotherapy combinations in breast cancer treatment are:

➢ FEC: Fluorouracil, Epirubicin, Cyclophosphamide

➢ FAC: Fluorouracil, Doxorubicine (Adriamycine), Cyclophosphamide

➢ TAC: Docetaxane, Doxorubicine, Cyclophosphamide

➢ EC - P (or EC - D): Epirubicine, Cyclophosphamide followed by either Paclitaxane or Docetaxane

➢ FEC-Doc: Fluorouracil, Epirubicine, Cyclophosphamide followed by Docetaxane

➢ TC: Docetaxane, Cyclophosphamide

➢ Formerly often applied CMF treatment regime (consisting of Cyclophosphamide, Methotrexate, and Fluorouracil) is nowadays more or less completely substituted by the above mentioned.

Thus, cisplatin at present does not play a pivotal role in clinical breast cancer therapy. However, Platinum-based chemotherapy could develop into a highly important new treatment modality with respect to yet incurable triple negative breast cancer (TNBC) [2]. Especially two TNBC subgroups seem to be amenable to Platinum-based chemotherapy: basal-like 1 and 2 (BL1, BL2). These two subgroups are identified by their Gene Expression Signature (GES) [3]. BL1 and BL2 subgroups of TNBC are characterized by high expression levels of DNA-damage response genes, which induce cell cycle arrest and apoptosis [2]. Interestingly, *in vitro *cell culture experiments unveiled this phenomenon and can possibly serve to predict the *in vivo *situation [2]. A different but also promising new idea is the use of PARP1 inhibitors as chemosensitisers in combination with Platinum-based chemotherapy. Preliminary results from clinical trials are promising and justify researchers hope for better clinical management of the disease in the near future as outlined in detail throughout this article.

## Platinum complexes as cytotoxic drugs

**Figure 1:**
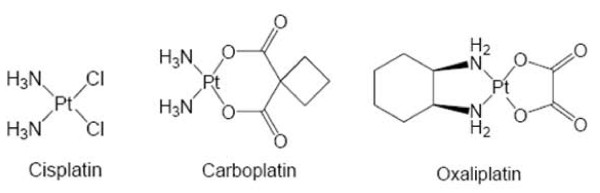
**Structure formulas of platinum-complexes**. Cisplatin, Carboplatin, and Oxaliplatin. Cis- and Carboplatin show high degree of cross-resistance, while oxaliplatin resistance seems to follow a different mechanism of action, showing only partial or no cross-resistance to Cis- and Carboplatin.

Cisplatin (Platinex^®^), Carboplatin (Carboplat^®^), and Oxaliplatin (Eloxatin^®^) (Figure 1) are first-line anti-cancer drugs in a broad variety of malignancies, for instance: ovarian cancer, testicular cancer and non small cell lung cancer. Cisplatin is inactive when orally administered and, thus, the prodrug Cisplatin must be toxicated endogenously. The active principle formed inside the cell is the electrophile aquo-complex. High extracellular chloride concentrations (~100 mM) prevent extracellular formation of the active complex. Upon entering the cell, in a low chloride environment (~2-30 mM), the aquo-complex is formed. The active principle is preferentially built as a shift in the reaction balance. The mechanism of action of the aquated complex at the molecular level is covalent cross-linking of DNA nitrogen nucleophils. The Cisplatin bisaquo-complex prefers an electrophilic reaction with N-7 nitrogen atoms of adenine and guanine. 1,2 or 1,3 intra-strand cross links are preferentially built (to an extent of about 90%). Affected are genomic and mitochondrial DNA molecules [4].

Carboplatin mechanistically acts similar to Cisplatin. However, a slower pharmacokinetic profile and a different spectrum of side effects has been reported [5]. The mechanism of action of Oxaliplatin substantially differs from Cis- and Carboplatin, which might be explained by the lipophilic cyclohexane residue. Cisplatin has a broad range of side effects. Problematic are nephro- and ototoxicity, but therapy-limiting is its extraordinary high potential to cause nausea and emesis. Thus, Cisplatin usually is administered together with potent anti-emetogens such as 5-HT_3 _antagonits (Ondansetrone, Granisetrone or else). Carboplatin has a diminished nephro- and ototoxicity, but can cause bone marrow depression, while oxaliplatins most characteristic side effect is dose-dependent neurotoxicity.

## Apoptosis attendant on DNA damage

Cytotoxic anti-cancer drugs excert their effect through the induction of apoptosis. The Greek derived word apoptosis (απόπτωσις) literally means *autumnally falling leaves*, describing a subject to be doomed. It is often refered to as programmed cell death. However, other mechanisms of programmed cell death have been identified recently, like autophagy, paraptosis, and mitotic catastrophe [6]. To this end, apoptosis more accurately is defined as cell death induced by caspases. Caspases are synthesized as inactive precursor proteins (procaspases) and activated upon proteolytic processing. They are divided into two major grous: (i) proinflammatory caspases (subtypes 1, 4, 5, 11, 12, 13, and 14) and (ii) proapoptotic caspases. Caspases triggering apoptosis are further categorized into initiating caspases (subtypes 2, 8, 9, and 10) and effector caspases (subtypes 3, 6, and 7) (reviewed in [7]).

Two apoptosis mediating pathways are divided, the intrinsic and the extrinsic apoptotic signaling pathway, with the latter induced by specific ligand-receptor interaction (for instance FasL - Fas interaction). The intrinsic apoptotic signaling cascade triggeres cell death induced by cytotoxic drugs. Accordingly, it is triggered among others by DNA damage [8]. This pathway is balanced by pro- and anti-apoptotic members of the Bcl-2 protein family. The tumour-supressor protein p53 is a pivotal point for the activation of the intrinsic apoptotic pathway: p53 responds to diverse cellular stresses by arresting cell cycle progression through expression of p53 target genes such as the mitotic inhibitors p27 and p21. After unrepairable DNA damage, p53 triggeres cell death via the expression of apoptotic genes (*puma*, *noxa*, etc.) and by inhibiting the expression of anti-apoptotic genes [9].

## Mechanisms of Cisplatin resistance

Cancer is one of the most deadly diseases world-wide with projected 1.596.670 new cases in 2011 in the USA alone [10]. Remarkable exceptions from this deadly rule are germ cell tumors of the ovary and testicular cancer when treated with cisplatin for which they show extraordinary sensitivity [11]. For testicular cancer cure rates of > 90% are reported after Cisplatin emerged as first line chemotherapeutic principle [12]. This is owed to the fact that testicular cancers do not develop Cisplatin resistance or cellular defense strategies against the drug. Chemotherapy is a central constituent for the treatment of cancer patients. However, cancer cells have the propensity to become resistant to therapy, which is the major limitation of current therapeutic concepts. Cancer patients usually are treated by repeated cycles of chemotherapy and the clinical course of most cancers is entailed with relapsed disease in the medium term. These recurrencies are paralleled by the development of therapy-refractory tumours representing a major problem in the clinical management of cancer patients. The emergence of chemoresistance is a time-dependent cellular process, which requires concerted action of many cellular components. Several mechanisms and pathways are involved in the emergence of a chemoresistant phenotype. Among others, general mechanisms of resistance known today are

• diminished drug accumulation

• elevated drug inactivation

• DNA repair or elevated DNA damage tolerance

• enhanced expression of anti-apoptotic genes, and

• inactivation of the p53 pathway (all reviewed in [4]).

However, this knowledge has not yet led to resounding clinical strategies to overcome cellular resistance: mechanisms of resistance are multiple and not all of them are fully understood. Specific principles of Cisplatin-resistance are reduced uptake or increased efflux of platinum compounds via heavy metal transporters, cellular compartimentation, detoxification of bioactive platinum aquo-complexes by Sulphur-containing peptides or proteins, increased DNA repair, and alterations in apoptotic signaling pathways (reviewed in [5]). Cisplatin and Carboplatin resistant cells are cross-resistant in all yet known cases. In contrast, Oxaliplatin resistant tumours often are *not *cross-resistant, pointing to a different mechanism of action. Cisplatin resistance occurs intrinsic (i.e. colon carcinomas [13]) or acquired (i.e. ovarian carcinomas [14]), but some tumour specimens show no tendency to aquire resistance at all (i.e. testicular cancer [12]). Reduced accumulation of Platinum compounds in the cytosol can be caused by reduced uptake, increased efflux, or cellular compartimentation. Several ATP binding cassette (ABC) transport proteins are involved like MRP2 and MRP6, Ctr1 and Ctr2, and ATP7A and ATP7B, respectively [15,16]. However, the degree of reduced intracellular Cisplatin accumulation often is not directly proportional to the observed level of resistance. This may be owed to the fact that usually several mechanisms of Cisplatin resistance emerge simultaneously. Another mechanism of resistance is acquired imbalance of apoptotic pathways. With respect to drug targets, chemoresistance can also be triggered by overexpression of receptor tyrosine kinases: ERB B1-4, IGF-1R, VEGFR 1-3, and PDGF receptor family members (reviewed in [17,18]). ERB B2 (also called HER 2) for instance activates the small G protein RAS leading to downstream signaling of MAPK and proliferation as well as PI3K/AKT pathway and cell survival. Experiments with recombinant expression of ERB B2 confirmed this mechanism of resistance. Meanwhile, numerous researchers are focussed on finding new strategies to overcome chemoresistance and thousands of publications are availible.

Another very recently discovered mechanism of cisplatin resistance is differential expression of microRNA. RNA interference (RNAi) is initiated by double-stranded RNA fragments (dsRNA). These dsRNAs are furtheron catalytically cut into short peaces with a length of 21-28 nucleotides. Gene silencing is then performed by binding their complementary single stranded RNA, i.e. messenger RNA (mRNA), thereby inhibiting the mRNAs translation into functional proteins. MicroRNAs are endogenously processed short RNA fragments, which are expressed in order to modify the expression level of certain genes [19]. This mechanism of silencing genes might have tremendous impact on resistance research. A very recently published article for instance focussed on differential microRNA expression in three cisplatin resistant germ cell tumour cell lines compared to their non-resistant, cisplatin sensitive counterparts [20]. The authors found a significant increase in the expression of a microRNA cluster (hsa-miR-371-373) in the cisplatin resistant situation, which triggeres p53 silencing [21]. Thus, a future perspective in the field of cisplatin resistance research might be to investigate microRNAs.

## Thiol-containing proteins and Cisplatin resistance

Among various mechanisms of platinum resistance, thiol-containing proteins are of special interest. Platinum-based complexes are the only heavy metal containing EMA- and FDA-approved cytostatics at present. This leads to a very uncommon possible mechanism of resistance: direct interaction of Cisplatin with thiol-groups forming a virtually insoluble sulphide. Since, this mechanism of action in resistance formation is exclusive to platinum-based compounds, it is referred to in this article with a special chapter.

Glutathione or metallothioneins are cysteine-rich peptides, capable of detoxicating the highly reactive aquo-complexes. Cisplatin resistance in ovarian cancer was reported directly proportional to increased intracellular glutathione [22]. However, increased glutathione levels are reversible but resistance is not. Upstream of gluthatione are further thiol-containing proteins called thioredoxins. Mammalian thioredoxins are a family of 10-12 kDa proteins characterized by a common active site: Trp-**Cys**-Gly-Pro-**Cys**. Thioredoxin-1 (TRX) is a 12 kDA ubiquitous protein of 104 amino acids with disulfide reducing activity [23]. TRX is frequently found in the cytoplasm, but was also identified in the nucleus of benign endometrial stromal cells, tumour derived cell lines, and primary tumours [24]. Its active site comprises two cystein residues in the consensus sequence serving as a general disulfide oxido-reductase. These two cystein residues (Cys-32, Cys-35) can reversably be oxidized to form a disulfide bond and be reduced by TRX reductase and NADPH [25]. The TRX system comprises TRX reductase, NADPH, and TRX itself. It is conserved throughout evolution from procaryotes to higher eucaryotes. The TRX system and the glutathione system constitute important thiol reducing systems [26]. TRX originally was identified as a hydrogen donor of ribonucleotide reductase in *Escherichia coli *[27]. Targeted disruption of the TRX gene in *Saccharomyces cervisiae *prolonged the cell cycle [28]. The TRX homologue gene of *Drosophila melanogaster *was identified as pivotal for female meiosis and early embryonic development [29]. The reducing nuclear environment, caused by thioredoxin, is preferable for the DNA binding activity of various transcription factors such as AP-1 [30], NF-κB [31], and the estrogen receptor [32]. AP-1 activation by TRX also occurs through an indirect mechanism: TRX reduces Ref-1, which in turn reduces cysteine residues within the fos and jun subunits of AP-1, thereby promoting DNA binding [30]. In the NF-κB molecule, TRX reduces Cys-62 of the p50 subunit in the nucleus, thereby allowing the transcription factor to bind DNA [33]. TRX in general regulates protein-nucleic acid interactions through the redox regulation of cystein residues [34]. In addition, cellular redox status is pivotal to regulation of apoptosis. TRX has been shown to bind and inactivate apoptosis signal-regulating kinase 1 (ASK1), with the latter to be released upon oxidative stress [35]. Apart from its cellular functions, TRX can be secreted as an autocrine growth factor by a yet unknown mechanism. It is then stimulating the proliferation of cells derived from a variety of solid tumors [36]. In addition, the cytochrom P450 subtype 1B1 (CYP1B1) converts 17β-estradiol (abbreviated as E2) into the carcinogenic 4-hydroxyestradiol (4-OHE2). A study conducted in ER-positive MCF-7 breast cancer cells suggested TRX to be involved in the constitutive expression of CYP1B1 and the dioxin mediated induction of CYP1B1 [37]. It may, thus, be a potent co-factor of mammary carcinogenesis at least in estradiol responsive tumours. Like other thiol-containing proteins, thioredoxin overexpression was suspected triggering chemotherapy resistance [24]. Hence, TRX overexpression in several tumour derived cell lines is associated with resistance to Cisplatin [38]. However, TRX effects on anti-cancer drug resistance are complex and depend strictly on the tissue type. For instance, hepatocellular carcinoma cells with elevated thioredoxin levels are resistant to Cisplatin, but not to the antracyclin Doxorubicin [39]. However, bladder- and prostate cancer cell lines with TRX overexpression are Cisplatin resistant and cross-resistant to Doxorubicin [40]. Cisplatin resistance in ovarian cancer cell lines is associated with high TRX levels, but recombinant TRX overexpression in non-resistant cells does not confer resistance to Cisplatin or Doxorubicin [41]. Thus, Cisplatin-responsiveness of a given tumour entity overexpressing TRX is unpredictable at present.

## Breast cancer

For midaged women in the industrialized countries, breast cancer is the second most common cause of cancer-death [10]. Carcinomas of the mammary gland comprise rather different diseases referring to divergent cell types found in the female breast. Breast cancers are divided into ductal, medullary, lobar, papillary, tubular, apocrine and adeno-carcinomas, respectively [42]. Breast cancer is not a purely gynecological disorder: approximately 1% of breast cancer cases are male patients. Apart from histological classification, breast cancers are biochemically categorized independent of the tissue origin with respect to their receptor status:

1. HER-2 positive tumours

2. triple-negative breast cancer (TNBC), which are ER, PR, and HER-2 negative

3. endocrine-responsive tumours

HER-2 positive tumours are characterized by constitutive overexpression of the HER-2 receptor subtype of the epidermal growth factor receptor family. Constitutive overexpression of HER-2 in invasive ductal carcinomas was reported in about 30% of all cases. On the one hand, HER-2 overexpression is a negative prognostic marker, on the other hand, HER-2 positive breast cancer can be targeted specifically, yielding an improved prognosis and fewer side effects [43]. No endogenous ligand for this receptor is known, but HER-2 has a fixed conformation that resembles the ligand activated state of the other HER subtypes [44]. In addition, HER-2 is the favoured dimerization partner of other ERBB receptors. HER-2 can be specifically targeted by means of humanized monoclonal antibodies Trastuzumab and Pertuzumab, respectively [18]. Both antibodies can also be administered over extended periods of time to avoid breast cancer relapse.

Triple negative breast cancer is not amenable to specifically targeted therapies, such as anti-hormone therapy or Trastuzumab. Therefore, classical chemotherapy is the only drug-based option in the therapeutic armamentarium at present [45]. In line with this, triple negative tumours carry a poor prognosis. TNBC accounts for approximately 15% of all breast cancer cases and younger (< 50 years) women are more frequently affected by TNBC than by HER-2 positive or hormone responsive tumours. It was recently discovered that the p53 family member p73 triggeres a pathway responsible for Cisplatin sensitivity in this subset of breast cancer specimens [46]. Thus, the authors suggested that these tumours could prevalently be treated with Cisplatin if stained positive for p73.

It is suggested that TNBC origins from BRCA1 or BRCA2 mutation carriers, since there is a 90% overlap between TNBC and BRCA mutation. Meanwhile, it is unveiled that BRCA mutations are often but not always associated with a triple negative phenotype [47]. However, especially BRCA mutated genotypes exhibit a Doxorubicine-sensitive [48] and Cisplatin-sensitive phenotype [49]. The reason is that DNA-damage affecting one allel cannot be compensated by homologous recombination because this would require an intact BRCA gene [50]. The impaired ability of homologous recombination is currently investigated in order to develop targeted therapy of BRCA mutation carriers. In BRCA mutated breast cancer patients, DNA-repair instead of homologous recombination is performed by Base Excision Repair (BER). In this context, a damaged nucleotide is excised and substituted by an intact nucleotide. This process requires (among others) the enzyme Polyadenosine 5'-Diphosphoribose Polymerase (PARP1). If PARP1 is inhibited in BRCA-mutated cells, both possibilities of DNA-repair are blocked [51]. This concept was tested recently with success in therapy-refractory Tumours with BRCA mutations. In this study, the oral bioavailable PARP1-inhibitor Olaparib (AZD2281) was applied. Treatment with Olaparib in a dose-escalation study caused stabe disease in 63% of cases [52]. Cisplatin as a directly DNA-interacting substance could be a drug of choice in combination therapy with Olaparib or any other PARP1-Inhibitor in BRCA-mutated breast cancer. Thus, PARP-inhibitors in the future could serve as chemo-senzitisers, which also was already successfully tested *in vitro *and *in vivo *[53,54].

The highest incidences have breast cancer specimens expressing the estrogen receptor, so-called hormone-responsive tumours. ER positive tumours are treated either with cytotoxic drugs, anti-estrogens or a combination of both. Anti-estrogens are estrogen receptor antagonists like Tamoxifen, Toremifen, Raloxifen or aromatase inhibitors blocking chemical transformation of Testosterone to the aromatic ring-A steroide Estradiol like Letrozole, Anastrozole. Since, pharmacologic inhibition is an additional treatment option in these cancer specimens ER expressing breast carcinomas carry a better prognosis than triple negative breast carcinomas. In line with this, the primary therapy approach usually shows good response. However, patients often face one or more relapses. The etiopathology of breast carcinomas often takes years, finally resulting in chemoresistant tumours. Chemotherapy triplets like FEC (comprising Fluorouracil, Epirubicin, and Cyclophosphamide) or CMF (Cyclophosphamide, Metothrexate, and Fluorouracil) are administered with the attempt to target multiple mechanisms of cancer cell mitosis and to avoid the emergence of resistance. However, after years or repeated chemotherapy cycles, the cancer cell finally aquires multiple resistancies [55]. Some of the applied substances (for instance Epirubicin) are outwardly transported by the membrane-spanning transport protein *plasmalemmal-glycoprotein*, 170 kDa P-gp (reviewed in [56]). Since, platinum-based compounds have no affinity towards P-gp, platinum based chemotherapy emerged in the recent years as second line treatment regimen for advanced breast cancer.

ER-positive breast cancers are the most prevalent form of the disease. Breast cancer patients with extensive lymph node involvement (advanced breast cancer) have a high disease recurrence rate. Eventually, in most women, metastatic breast cancer becomes refractory to hormonal treatment and chemotherapy [57]. These findings demonstrate that the development of resistance to therapy is a long term clinical process. During our studies we have generated Cisplatin resistant ER-positive breast cancer cells (MCF-7 CisR) by sequential cycles of Cisplatin exposure over a period of 6 months. During the first two months the cells received weekly cycles of Cisplatin followed by monthly cycles of Cisplatin exposure. We used these cells to investigate systematically the activities of various signalling networks, comprising ERBB and MAPK signaling pathways using phospho-proteome profiling. In MCF-7 CisR cells the EGFR is phosphorylated. Downstream we found Both, MAPK and PI3K/AKT kinase activation with AKT kinase being reported to mediate chemoresistance in breast cancer cells. In line with this, inhibition of AKT-kinase activation by pharmacological tools in MCF-7 CisR cells was entailed with reversal of Cisplatin resistance. In addition, AKT kinase up-regulates *Bcl-2 *expression with BCL-2 preventing apoptosis independent of the structure of the causing drug [58].

The EGFR pathway is activated by an array of ligands binding the four EGFR receptor monomers in divergent composition [18]. These ligands can act in form of an autocrine loop in self-sufficient cancer cells. In our study, gene expression profiling and RT-PCR revealed that EGFR-ligand amphiregulin is overexpressed and secreted in resistant MCF-7 cells. Amphiregulin is an exclusive ligand of the EGFR which induces tyrosine trans-phosphorylation of EGFR-dimerized subunits leading to subsequent receptor activation [59]. Amphiregulin originally was purified from the conditioned media of MCF-7 cells treated with the tumour promoter PMA [60]. Amphiregulin increases invasion capabilities of MCF-7 breast cancer cells, and transcriptional profiling experiments revealed that amphiregulin promotes distinct patterns of gene expression compared to EGF [61]. Several genes involved in cell motility and invasion are upregulated when nontumourigenic breast epithelial cells are cultivated in the presence of amphiregulin. The cytoplasmic tail of the EGFR plays a critical role in amphiregulin mitogenic signaling but is dispensable for EGF signaling [62]. Autocrine loop formation leading to independence of extrinsic proliferative signals is a key event in the evolution of malignant tumours. In our study, we found a significantly increased ability to invade and penetrate the basement of the matrigel invasion assay. These results are in line with published data and they show that drug resistance and tumour aggressiveness are interconnected processes. As a proof of principle, this consideration was tested by amphiregulin knock down experiments. It was possible to overcome Cisplatin resistance to a large part by siRNA mediated knockdown of amphiregulin gene expression. Amphiregulin protein is anchored to the cell membrane as a 50-kDa proamphiregulin precursor and is preferentially cleaved by ADAM 17 at distal site within the ectodomain to release a major 43-kDa amphiregulin form into the medium [63]. We conclude that MCF-7 cells show persistant alterations of signaling activity in the ERBB pathway associated with an inactivation of p53 and BCL-2 overexpression.

**Figure 2:**
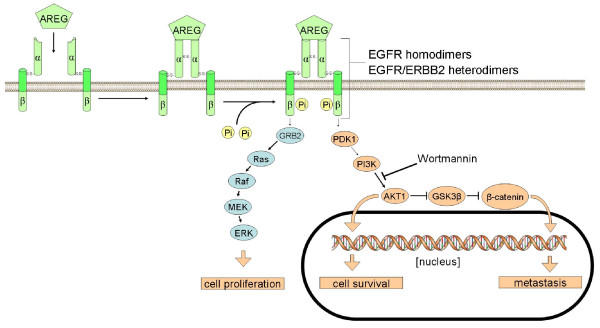
**Schematic model of Amphiregulin signalling**. Amphiregulin induced signaling of the EGFR/ERBB2 receptor tyrosine kinases in Cisplatin resistant MCF-7 cells.

An overview of the biochemical mechanisms underlying Cisplatin resistance in MCF-7 breast cancer cells is given in Figure 2. Once a molecular mechanism is unveiled it is mandatory to explore whether this finding is a general mechanism. To address this issue we correlated amphiregulin expression levels with the Cisplatin resistant state of a collection of human breast cancer cells and found a correlation which demonstrates that breast cancer cells use amphiregulin as a survival signal to resist exposure to Cisplatin [64]. We also analyzed a collection of lung cancer cells which tend to express elevated levels of amphiregulin, too. In contrast to breast cancer cells, a correlation between Cisplatin resistance and amphiregulin expression in lung cancer cells was not detected. Thus, it is necessary to investigate different tumour types and stages in order to determine the role of amphiregulin for Cisplatin resistance. Further studies will determine the impact of amphiregulin expression for therapy response and outcome in women with breast cancer.

## Ovarian cancer

Clinicians have designated ovarian cancer a "silent killer" because, when diagnosed, the disease usually has already spread into the peritoneum [65]. If the cancer is diagnosed while confined to the ovary (localized stage), the 5-year survival rate is over 90%. In contrast, if ovarian cancer is diagnosed after it has metastasized (distant stage), the 5-year survival rate is below 30%. Unfortunately, most cases (68%) are diagnosed at the distant stage. Thus, ovarian cancer has a substantially shorter and more dramatic etiopathology than breast cancer: ovarian cancer is the most lethal gynecological cancer in the industrialized nations although its first occurrence has a satisfactory clinical response to platinum-based chemotherapy [10]. The reason is that more than 80% of the patients experience an early relapse [66]. The tumour usually reappears in advanced stage or as metastatic form of the disease (FIGO III/IV), which is treated in first line with cytoreductive surgery followed by chemotherapy doublets consisting of a Platinum-based compound combined with a Taxane. Resistance to Platinum-containing compounds is a major obstacle in ovarian cancer therapy and the underlying mechanisms are not completely understood. Formation of a Cisplatin resistant phenotype after initial drug response usually is entailed with a lethal course of the disease after a relapse [67]. Cellular defense to Cisplatin evolves as concerted action of growth factors, RTKs, MAPKs and other signal transduction pathways. The emergence of ovarian cancer proceeds with clinically diffuse symptoms [68]. Unfortunately, ovarian cancer is not contemporarily diagnosed because early symptoms like abdominal pain are not regarded as signs of a deadly disease by the patient. When symptoms aggravate, the patient often is already moribund. Ovarian cancer incidence peaks in the sixth and seventh life decade [67]. Approximately 5% of ovarian cancer cases have a hereditary background: women bear an increased risk of ovarian cancer if a first-degree relative suffers from (or died of) ovarian or breast cancer [69].

Therapeutic intervention of ovarian carcinomas can have different intentions, first, a curative approach intending the complete removal of the tumour and significant extension of survival time. To achieve this objective, severe side effects are accepted. Second, palliative therapy intends to enhance patient's quality of life and to alleviate pain and other disease symptoms. In the latter case, aggressive treatment options are avoided. Regarding chemotherapy, adjuvant and neo-adjuvant regimens are used: in an adjuvant chemotherapy regimen, cytostatic drugs are given *after *a debulking surgery, whereas in a neo-adjuvant setting, cytostatic drugs are given *prior *to cytoreductive surgery. The intention of adjuvant chemotherapy is to eliminate remaining tumour cells, thereby, preventing a relapse. Neo-adjuvant chemotherapy aims at reducing the tumour burden before surgery, intending to remove the tumour completely with one large surgery [70].

The crucial step in ovarian carcinoma treatment is the first surgery of the primary tumour, since only this can cure the disease [71]. All regimens applying chemotherapy (at present) are only of palliative value. The current standard chemotherapy comprises a combination of Carboplatin and Paclitaxel. Alternatively, a combination of Carboplatin and Gemcitabine may be used. However, the majority of patients will face relapsed disease. Approximately 20% are Platinum-refractory early relapses with very poor prognosis occuring within the first 6 months after therapy. The remaining 80% are Platinum-sensitive late relapses. In the first case, Topotecan or the antracycline Doxorubicin, masked in liposomes of polyethylenglycol, are considered as a remaining therapy option. In the latter case (Platinum-sensitive relapse) a Carboplatin/Paclitaxel doublet remains first choice chemotherapy. Therapy of relapsed ovarian cancer always is of palliative nature, thus, intending to delay disease progression, reduce pain, and maintain quality of life [67].

Clinical findings show that the development of resistance to therapy of ovarian cancer is a time-dependent biological process [65]. In our study we used A2780 epithelial ovarian cancer cells as a model system to investigate the molecular determinants of Cisplatin resistance and uncovered the molecular mechanism of action. Since A2780 is not a representative cell line for the most common histology subtype of epithelial ovarian cancer, we generalized our findings by analysing also HEY, OVCAR-8, SKOV-3, and BG-1 cell lines. In addition, a clinical trial with 80 ovarian cancer tumour samples was analysed. To mimic the clinical situation of Cisplatin therapy *in vitro*, we followed the same procedure as with MCF-7 breast cancer cells: we generated Cisplatin-resistant cells by weekly cycles of Cisplatin at a dose, which is reached in patients in the clinic and assessed the emergence of resistance during 6 months. We found a correlation of increasing *IGF-1R *mRNA expression levels with the emergence of resistance to Cisplatin. In order to analyse generalisability of this finding, we correlated *IGF-1R *mRNA expression with the intrinsic Cisplatin resistance status in a panel of human ovarian cancer cells and found a significant correlation [72]. The IGF-1 receptor is physiologically expressed in the ovary and it was reported that its pathway is functional in human ovarian surface epithelial cells which are the origin of most epithelial ovarian carcinomas [73,74]. It is, therefore, not surprising that nearly all ovarian carcinomas and ovarian cancer-derived cell lines express the IGF-1 receptor at the cell surface [75]. The IGF-1 receptor pathway regulates many processes in ovarian epithelial cells [76]. Hyperactivation in our model system is explained by an IGF-1 based autocrine loop. IGF-1 is a multifunctional peptide of 70 amino acids. Upon binding to the IGF-1R the ligand activates the IGF-1R tyrosine kinase function. After mutual phosphorylation of the β-subunits (Y 950, Y 1131, Y 1135, Y 1136), the active receptor phosphorylates the adaptor protein insulin receptor substrate (IRS-1) at S 312. This leads to either complex formation with a second adapter protein, GRB-2, and activation of the guanine nucleotide exchange factor SOS resulting in RAS/RAF/MEK/ERK activation, or direct activation of PI3 kinase [77]. Class I PI3Ks are divided into two subfamilies, depending on the receptors to which they couple. Class IA PI3Ks are activated by RTKs, whereas class IB PI3Ks are activated by G-protein-coupled receptors [78]. Class IA PI3Ks are heterodimers of a p85 regulatory subunit and a p110 catalytic subunit. Class IA PI3Ks regulate growth and proliferation downstream of growth factor receptors. It is, thereby, interesting to note that the IGF-1 receptor primarily regulates growth and development and has only a minor function in metabolism [79].

A recent report has shown that coactivation of several RTKs in glioblastoma obviates the use of single agents for targeted therapies [80]. Fortunately, in our model system of Cisplatin resistant ovarian cancer, we did not detect coactivation of other RTKs besides IGF-1R. To further analyse this, we functionally inactivated IGF-1 in tissue culture supernatants which caused a reversion of the Cisplatin-resistant phenotype. Likewise, inhibition of IGF-1R transphosphorylation and signaling by small molecule inhibitors had a similar effect.

We and many other researchers have demonstrated that signaling through PI3K pathway provokes Cisplatin resistance in ovarian cancer. In addition, reports from the literature show that PI3K signaling is important for the etiology of ovarian cancer. It is well established that AKT signaling plays a major role for cell survival (reviewed in [81]). However, AKT isoforms can have different functions as it was shown that AKT1 is required for proliferation, while AKT2 promotes cell cycle exit through p21 binding [82]. The *AKT2 *gene is overexpressed in about 12% of ovarian cancer specimens, which indicates that it may be linked to the etiology of the disease [83]. However, AKT2 has also been linked to the maintenance of a Cisplatin resistant phenotype of ovarian carcinomas: it was shown that AKT2 inhibition re-sensitized Cisplatin resistant ovarian cancer cells [84]. In our study, an expression profiling from 80 ovarian carcinomas unveiled the regulatory subunit *PIK3R2 *as a negative prognosis factor for ovarian cancer. This result is in line with the findings of an independent study by Dressman and coworkers [85].

## Common features of Cisplatin resistance models

Table 1 summarizes the key findings of our studies in gynaecological cancer *in vitro *models of Cisplatin resistance.

**Table 1:** Comparison of Cisplatin resistance in vitro models of A2780 ovarian cancer cells and MCF-7 breast-cancer cells

	altered in Cisplatin resistant	
**Read-out**	**MCF-7 CisR**	**A2780 CisR**

*Cisplatin resistance factor*	3.3***	5.8***

proliferation rate [%]	192**	55.3***

*invasive capacity [%] compared to parental cells*	153.7*	129.5*

*RTK activation in Cisplatin resistant cells*	*EGFR/ERB-B2*	*IGF-1R*

autocrine growth factor	amphiregulin	IGF-1

bystander effect	no	IGF-1 mediated

ERK1,2 activation	elevated	elevated

p38 activation	no	p38α

JNK activation	no	no

AKT kinase activation	elevated	elevated

An overview of the long-term functional and biochemical changes after establishment of Cisplatin resistance is given. Cisplatin resistant breast cancer cells and ovarian cancer cells were compared to their non-resistant parental cells. Denoted are the changes observed in the Cisplatin resistant situation [64,72].

It is evident that both models exhibit elevated invasiveness and specific growth factor receptor activation exclusively in the Cisplatin resistant situation (red labeled in table 1). However, the activated class of RTKs differs in the tumor entities. Cisplatin resistant

(i) breast cancer cells show EGFR/ERBB2 activation

(ii) ovarian cancer cells show IGF-1R activation

At first sight, these tumour entities seem to follow different biochemical mechanisms to archieve a similar functional outcome, which is downstream activation of the PI3K/AKT-pathway. However, these biochemical signaling routes converge at a single axis: the estradiol/estrogen receptor activation, which is the decisive route in female organ ontogenesis. With respect to developmental processes of the respective tissue, the activated receptors in the Cisplatin resistant state are of high ontogenic importance. Ontogenesis of the female primary and secondary sexual organs are divided into two phases with an intermediate quiescence period of 10-15 years: (i) prenatal organ development and (ii) puberty, resulting in a functioning reproductive system at the time of menarche.

## Conclusions

At first sight it seems a paradoxon that a mechanism inducing proliferation (amphiregulin) triggeres Cisplatin resistance. A fast growing cell presents a better target for classical chemotherapeutic drugs. However, both differentially activated RTKs, ERGF and IGF-1R, not only signal through the MEK/ERK pathway, resulting in enhanced proliferation responses, but also through the PI3K/AKT survival pathway. Many of the signaling molecules downstream of the receptors are identified as oncogenes, like ras- or raf small G proteins. Therefore, these factors can be looked at as a two-edged sword: with the eyes of a developmental biologist they are pivotal in ontogenesis; with the eyes of a tumour biologist, they can trigger oncogenic transformation and concomitantly resistance to chemotherapy. Since, the PI3K/AKT pathway is a general apoptosis preventing pathway, resistance is triggered not only to a special group of drugs but towards chemotherapy as a whole. This is supported by the finding that the Cisplatin-resistance models in our studies showed cross-resistance towards Doxorubicine, an anti-cancer drug, which is chemically unrelated to Cisplatin. Therefore, resistance-mediating factors derived from proteins with prominent function in organ ontogenesis could be designated as "resistogenic".

## List of abbreviations used

RTK: receptor tyrosine kinase; TKI: tyrosine kinase inhibitor; EGFR: epidermal growth factor receptor; HER-2: Human epidermal growth factor receptor type 2; IGF-1R: insulin-like growth factor receptor: PDGFR: platelet derived growth factor receptor; bbb: blood brain barrier; P-gp: P-glycoprotein; TRX: thioredoxin; MAPK: Mitogen-activated protein kinase; CDK: cyclin-dependent kinase; ER: estrogen receptor; PR: progesterone receptor; TNBC: triple negative breast cancer; P-gp: plasmalemmal-glycoprotein; PMA: Phorbol-Myristate-Acetate; ADAM: a disintegrine and metalloproteinase; IRS-1: Insuline receptor substrate;

## Competing interests

The authors declare that they have no competing interests.

## Authors' contributions

not applicable

## Acknowledgements

Critically reviewing of the manuscript by Dr. Bodo Haas is greatfully acknowledged. This review article was supported by intramural funding of the Federal Institute for Drugs and Medical Devices.
